# Endoparasites in carnivores in Swiss zoological institutions between 2009 and 2024: evaluation of risk factors and deworming strategies

**DOI:** 10.1016/j.ijppaw.2026.101199

**Published:** 2026-01-27

**Authors:** Jasmin Tan, Sarah Weber, Ronja Zuber, Maya Kummrow, Manuela Schnyder

**Affiliations:** a Institute of Parasitology, Vetsuisse Faculty, University of Zurich, Winterthurerstrasse 266a, 8057, Zurich, Switzerland; b Clinic for Zoo Animals, Exotic Pets and Wildlife, University of Zurich, Winterthurerstrasse 260, 8057, Zurich, Switzerland

**Keywords:** Zoo carnivores, Switzerland, Intestinal and respiratory parasites, Clinical observations, Risk factors, Management measures

## Abstract

Parasitic infections in zoo carnivores pose risks to animal health and zoonotic transmission. Over a 15-year period, endoparasites were detected in faecal samples from Swiss zoo carnivores, with clinical and management data used to identify factors linked to parasite occurrence. The samples, originating from 50 species kept in 30 different institutions, were collected in 2009 (n = 140), 2018 (n = 149) and 2024 (n = 116), and analysed by Baermann-Wetzel technique, combined sedimentation/flotation, *Giardia* coproantigen detection and Ziehl-Neelsen staining of faecal smears. Fifteen samples were further analysed by biomolecular methods. Odds ratios and 95 % confidence intervals (OR, 95 % CI) were calculated to compare management strategies and assess potential risk factors. Overall, 198/405 (48.9 %) of the samples tested positive. The most frequent parasites were ascarids (n = 73), followed by *Capillaria* spp. (n = 66), *Cystoisospora* spp. (n = 50) and *Cryptosporidium* (n = 43). Most animals were asymptomatic. Among the animals with clinical observations available, diarrhoea was recorded in ten, respiratory signs in two and reduced body condition in four individuals. Of these, intestinal (e.g., ascarids, hookworms, *Cystoisospora* sp., *Cryptosporidium* sp.) or respiratory (e.g., *Angiostrongylus vasorum*, *Capillaria boehmi*) parasites were identified in seven animals. Hunting mice, birds, rodents or eating snails was significantly associated with positive findings (OR: 2.3, 95 % CI: 1.0–5.0, p = 0.04), whereas on-site slaughter of food animals appeared protective (OR: 0.5, 0.2–1.1, p = 0.09). Zoonotic parasites such as *Strongyloides* sp. (n = 3) and *Baylisascaris* sp. (n = 3) were rarely detected. Animals from institutions that did not apply any parasitological management measures (deworming and/or faecal examinations) were more likely to be positive for helminths compared to those from zoos applying such measures (OR: 3.4, 95 % CI: 1.1–10.3, p = 0.03). Although often subclinical, some parasites—especially respiratory helminths—can affect animal health, and stress or anaesthesia may exacerbate diseases in valuable individuals. Targeted, diagnosis-based deworming reduces anthelmintic use, and consequently the risk of resistance and the likelihood of adverse effects.

## Highlights

• We analysed faecal samples of 50 zoo carnivore species from 30 Swiss institutions.
• Over a 15-year period, 198/405 (48.9 %) of samples tested parasitologically positive.
• Clinical signs observed in 16 animals; associated parasites in seven of them.
• Hunting preys or eating snails was significantly associated with positive findings.
• On-site slaughter of food animals appeared protective (OR: 0.5, 0.2–1.1, p = 0.09).

## Graphical abstract

**Figure:**
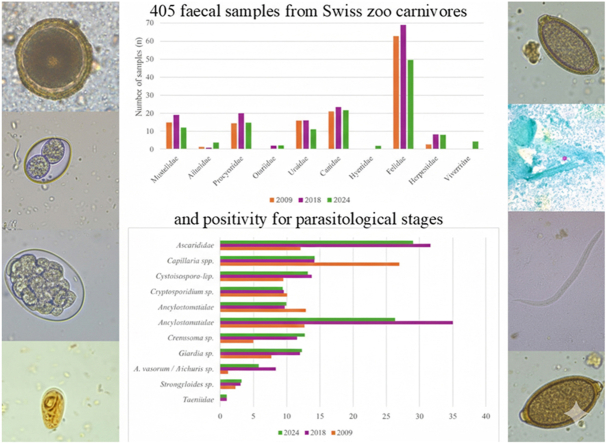

## 1 Introduction

Wild carnivores are kept in zoos and wildlife parks for educational, recreational and scientific purposes as well as for species conservation (Conde et al., 2011; Roe et al., 2014). For zoo animals, the enclosure habitat often remains the same over years, if not decades, which facilitates the accumulation of parasitic stages in their environment. Accordingly, prevention and/or management of intestinal and extraintestinal parasite infections represents a major challenge when keeping zoo carnivores (Panayotova-Pencheva, 2013).

Worldwide, the prevalence of intestinal parasites in zoo carnivores varies from 12.5 % (Dhakal et al., 2023) to 65.3 % (da Silva Barbosa et al., 2020), 67.0 % (Taki and Bourquia, 2023) up to 90.6 % (Shemshadi et al., 2015), reflecting high variability associated with local differences in the parasitic spectrum as well as in the specific management situation, which influence the parasite loads and environmental contamination. In a broader comparison among taxonomic orders, endoparasite prevalence in zoo animals in Germany has recently been described as highest in animals of the orders Artiodactyla and Perissodactyla, followed by Carnivora (Murnik et al., 2024). In a survey on carnivores kept in a Malaysian zoological garden, high prevalences (89.3 %) have been particularly reported for felids (Lim et al., 2008).

In Europe, ascarids were described as the most frequently detected helminths, occurring e.g. in 17 % of carnivore samples from 41 zoos and wildlife parks in Eastern Germany (Murnik et al., 2024), and in two-thirds of examined felid samples from Italian parks and from a Polish zoo (Fagiolini et al., 2010; Maesano et al., 2014). In contrast, protozoan infections were less frequently detected, with approximately 10 % positive samples (Fagiolini et al., 2010; Murnik et al., 2024).

Metastrongyloid lungworms such as *Angiostrongylus vasorum, Crenosoma* spp. and *Aelurostrongylus abstrusus* cause severe clinical signs in infected dogs and cats (Deplazes et al., 2016), and clinical cases with partially fatal outcomes have also been described in zoo and wild carnivores (Bertelsen et al., 2010; Deak et al., 2023; De Liberato et al., 2017; Gillis-Germitsch et al., 2017; Mahjoub et al., 2020; West et al., 1977). Among these parasites, especially *A. vasorum* shows an increasing prevalence in wild foxes throughout Europe, including Switzerland (Gillis-Germitsch et al., 2020b). Due to their invasiveness and prominent presence in cities and on zoo premises, foxes represent a prominent source for direct and indirect (through intermediate hosts) environmental contamination with this and other parasites for zoo canids.

Further risk factors leading to high burdens of endoparasite in zoo-housed mammals have been described. Feed selection, environmental conditions and management measures such as quarantine before introducing new animals play a role in the occurrence of parasite infections (Panayotova-Pencheva, 2013). However, the high variability of parasitic life cycles, which often include intermediate and/or paratenic hosts, as well as the different prepatent periods and survival rates of parasitic stages (depending on factors such as temperature and humidity), contribute to a highly complex situation that needs consideration in the specific zoo setting.

There is limited literature on specific risk factors for zoo carnivores associated with health issues due to endoparasite infections (Esteban-Sánchez et al., 2024).

The present study focuses on the occurrence of intestinal and lung parasites in most of the zoo carnivores present in Swiss wildlife parks and zoos, and in three different years of investigation. The data were complemented with information on the animals’ health, where available, and the respective zoo management. The aim of the study was to identify potential risk factors and to develop standardised recommendations for endoparasite management in zoo carnivores.

## 2 Material and methods

### 2.1 Study population and faecal sampling

Zoos and wildlife parks of Switzerland with known husbandry of wild carnivores were contacted in three different time periods and invited to take part in the study. In 2009, all 23 contacted zoos and wildlife parks participated, and a total of 140 samples were analysed. The second time, between 2017 and 2018, 24 out of 27 zoos agreed to participate, and a total of 149 samples were analysed. Additionally, three private keepers of wild felids were included, with one sample each. The third time, in 2024, 22 out of 29 contacted zoos and wild parks participated, and a total of 116 samples were analysed. For the last study period in 2024, the responsible zoo staff was asked to only send samples when animals had not been dewormed for two months. Sampling was either done by animal keepers or the researchers themselves. More than half were one-day samples: 54.0 % one-day samples (n = 143), 14.3 % two-day samples (n = 38), 30.9 % three-day samples (n = 82) and 1.1 % (n = 3) four-day samples (information was not available for samples from 2009 and two samples from 2018). Whenever possible, researchers visited the institutions and brought the faecal samples directly to the laboratory of the Institute of Parasitology, University of Zurich, Zurich, Switzerland. Upon arrival, the samples were either directly examined or stored at 7 °C and analysed within a maximum of two days.

### 2.2 Questionnaire

In all three years, supplementary data were collected on the number and age of the animals investigated, husbandry practices and deworming schemes. Data of animals from overall ten families, i.e., Mustelidae, Ailuridae, Procyonidae, Otariidae, Ursidae, Canidae, Hyenidae, Felidae, Herpestidae and Viverridae with respectively 10, 1, 3, 1, 8, 7, 1, 14, 3 and 2 different animal species were collected (for details, see Suppl. file 1 and 3). This was done in most detail in 2024, with an oral interview being conducted on-site in each facility. Data on potential risk factors such as feeding (i.e., on-site slaughter of food animals, spring water availability), characteristics of the enclosures (i.e., opportunity for catching wild animals, observed presence of mice and/or slugs in the enclosures), deworming management (divided in the following categories: 1 = no deworming, no strategy; 2 = deworming frequency <4x/year; 3 = deworming at least 4x/year; 4 = targeted deworming following positive faecal examinations, conducted less than four times annually; 5 = targeted deworming following positive faecal examinations carried out 4x/year or more) and previous findings related to parasitological examinations (if available) were collected in an excel file (Suppl. file 1).

### 2.3 Faecal analyses

A combined sedimentation/flotation method using zinc chloride as a flotation solution (density: 2.91 g/cm^3^) and the Baermann-Wetzel funnel method were performed with 10 g of faeces each (Deplazes et al., 2016). Furthermore, faecal smears of all samples were stained with Ziehl-Neelsen staining for *Cryptosporidium* detection, and a commercial coproantigen detection ELISA (RIDASCREEN® *Giardia*, R-Biopharm, Germany) was used for *Giardia* antigen detection.

### 2.4 Molecular analyses

Five microscopically positive findings for helminths of the respiratory tract as well as ten samples positive for potentially zoonotic parasites (i.e., *Cryptosporidium* sp., taeniids, *Strongyloides* sp. and *Baylisascaris* sp.) from 2018 to 2024 were further investigated and confirmed by molecular analyses. Samples originating from Baermann funnel method and the faecal sediments of flotation procedures were subjected to freeze-thaw cycles in liquid nitrogen. Sediments were then resuspended in 200 μl of PBS to ensure a more homogeneous mixture. DNA was isolated with the QIAamp Tissue DNA Mini Kit (Qiagen, Hilden, Germany) following the manufacturer's instructions, and stored at – 20 °C.

Samples positive for first stage larvae (L1) of *A*. *abstrusus* were further analysed applying a duplex PCR to correctly distinguish them from L1 of *Troglostrongylus brevior*. The duplex PCR protocol amplifies the ITS-2 gene region of respectively *A. abstrusus* (220 bp) and *T. brevior* (370 bp) (Annoscia et al., 2014). The described protocol was performed with the following modifications: the reaction volume (50 μl) contained 25 μl Mastermix (Qiagen multiplex PCR kit), 18 μl nuclease-free water, 0.5 μl (1uM) of AeluroF and TrogloF, 1 μl (2uM) of MetR-rc and 5 μl of genomic DNA template or nuclease-free water as a negative control. The amplification protocol used was the following: 95 °C for 5 min (for polymerase activation); followed by 40 cycles of 95 °C for 30 s (denaturation), 50 °C for 30 s (annealing), 72 °C for 30 s (extension); followed by 10 min at 72 °C (final extension).

For confirmation of positive samples for L1 of *A*. *vasorum* a 180 bp fragment of the ITS2 gene was amplified using primer pairs previously described (Jefferies et al., 2011). The PCR reaction was prepared in a final volume of 50 μl, consisting of 25 μl Mastermix (Qiagen multiplex PCR kit), 19 μl nuclease-free water, 0.5 μl of each primer I2F10 and I2R9 (1uM) and 5 μl of genomic DNA template or nuclease-free water as a negative control. The amplification protocol was modified as follows: 94 °C for 10 min followed by 40 cycles of (95 °C for 30 s, 62 °C for 30 s and 72 °C for 30 s), followed by 72 °C for 10 min. For the above-mentioned protocols, sequencing of the positive samples was not performed for species confirmation, as the presence of amplicons of the expected size was considered sufficient to identify the parasite species.

Faecal smears showing morphologically unclear results for *Cryptosporidium* were further examined using a PCR targeting a 420 bp fragment of the 18s rDNA (Ward et al., 2002). The reaction volume (50 μl) contained 25 μl Mastermix (Qiagen multiplex PCR kit), 19 μl nuclease-free water, 0.5 μl of CPB-DiagF and PW99R each (1uM) and 5 μl of genomic DNA template or nuclease-free water as a negative control. The amplification protocol was modified to the following: 94 °C for 10 min; 40 cycles of 94 °C for 30 s, 60 °C for 30 s, 72 °C for 45 s; followed by 10 min at 72 °C. A sample was considered positive for *Cryptosporidium* if tested positive by either Ziehl–Neelsen staining or if positive by PCR. Smears with ambiguous staining results that were PCR-negative were classified as negative. Amplicons were not sequenced, as the PCR was used only to confirm unclear smear results.

To determine the species of *Baylisascaris* in positive samples, a genus-specific PCR targeting a 146 bp fragment of the cox2 mitochondrial gene was performed (Dangoudoubiyam et al., 2009), with modifications. A 50 μl volume consisting of 25 μl Mastermix (Qiagen multiplex PCR kit), 19 μl nuclease-free water, respectively 0.5 μl of primer BpF and BpR (1uM), and 5 μl of genomic DNA template or nuclease-free water as a negative control was used. The amplification protocol was slightly modified to 94 °C for 10 min followed by 40 cycles of (95 °C for 1 min, 48 °C for 30 s and 72 °C for 30 s), followed by 72 °C for 10 min.

Morphologically positive samples for *Strongyloides* spp. larvae or eggs were further genetically analysed to identify the species. A previously described protocol (Labes et al., 2011) targeting a 270 bp fragment of the 18S rRNA was used, with the following modifications: the reaction volume (50 μl) contained 25 μl Mastermix (Qiagen multiplex PCR kit), 19 μl nuclease-free water, 0.5 μl of each StrongoF and StrongoR (1uM) and 5 μl of genomic DNA template or nuclease-free water as a negative control. The following amplification protocol was used: 94 °C for 10 min; 40 cycles of 94 °C for 30 s, 58 °C for 30 s, 72 °C for 30 s; followed by 72 °C for 10 min.

Samples containing taeniid eggs were subjected to further species-level identification using a multiplex PCR method based on mitochondrial gene targets (Trachsel et al., 2007).

The amplicons obtained from the latter three PCR protocols were directly sent for sequencing. DNA was purified using MinElute PCR purification kit (Qiagen, Hilden, Germany). Sequencing was performed by Microsynth (Microsynth AG, Switzerland https://www.microsynth.com/address.html), and sequencing results were analysed by comparing them with entries in the GenBank database using the BLAST search tool (http://www.blast.ncbi.nlm.nih.gov).

### 2.5 Statistical analyses

For statistical evaluation, the R Project for Statistical Computing (RStudio, The R Foundation for Statistical Computing, Vienna, Austria) as well as Microsoft Windows Excel 2007 were used. Chi-square test (with eight or more animal species examined) and Fisher exact test (with fewer than eight species) were used to assess the distribution of endoparasitic infection across animal species. P values of *P* ≤ 0.05 were considered statistically significant. Exact binomial 95 % confidence intervals (CI) for means of binomial variables were calculated according to Clopper and Pearson (1934). Associations between the detection of parasitic stages in faeces and the observation of clinical signs, deworming management, and potential risk factors such as on-site slaughter, spring water availability, the opportunity for catching wild animals in the enclosures, as well as for the observed presence of mice and/or slugs in the enclosures were calculated using odds ratios (OR), supplemented with the 95 % Confidence Intervals (95 % CI), calculated with MedCalc Software Ltd. Odds ratio. https://www.medcalc.org/en/calc/odds_ratio.php (Version 23.3.7; accessed October 5, 2025).

## 3 Results

### 3.1 Questionnaire

**Fig. 1:**
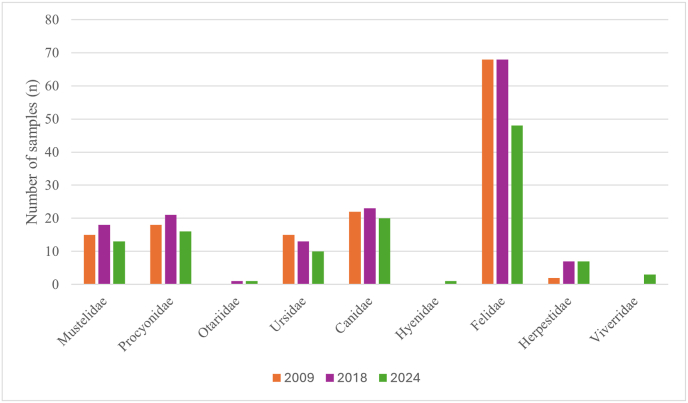
Number of examined faecal samples/year per carnivore family collected from 50 carnivore species kept in 30 zoos and wildlife parks of Switzerland examined in 2009, 2018 and 2024.

In 2009, 2018 and 2024, 23, 24 and 22 zoos participated in the study, respectively: 15 zoos were sampled in all three years, and a further eight zoos were sampled twice. Geographically, very few zoos were located above 700 m above sea level (6/23 in 2009, 2/24 in 2018 and 4/22 in 2024). Overall, 50 animal species belonging to ten carnivore families were included, with 140 samples in 2009, 149 samples in 2018 and 116 samples in 2024 (Fig. 1). Samples were collected between August and October (2009 and 2024) or between June 2017 and April 2018.

Most samples were pooled samples obtained from more than one animal in the same enclosure (77.9 % in 2009, 61.8 % in 2018 and 80.2 % in 2024). Most were obtained from adult individuals only, complemented with 18.6 % (2009), 14.3 % (2018) and 15.5 % (2024) originating from juveniles (defined as animals before sexual maturation), or from adult individuals kept together with juveniles. Where information was available, the animals were mostly born in captivity.

Overall, most zoos and wildlife parks adopted specific strategies to prevent parasite infections (e.g., deworming with or without previous faecal examination), and an increase in the number of zoos with a defined strategy was observed over time. In 2009, 14 out of 23 zoos had a strategy; however, no details were collected. In 2018, 18 out of 24 zoos or private collections reported implementing strategic parasite control measures. Approximately half of these (n = 8) conducted routine faecal examinations and administered anthelmintics based on positive findings, while the remainder followed various deworming schedules. Of the six remaining institutions, two had no strategy in place, and data were not available from four. In 2024, all zoos had adopted deworming management strategies, with 16 out of 22 zoos adopting targeted deworming measures depending on the coproscopic results. For 2018 and 2024 (with more detailed information), the adopted deworming strategies were evaluated in relation to positive or negative findings of helminth stages. Samples that tested positive for protozoa only were classified as ‘not infected with helminths’. Overall, when comparing the different management and deworming strategies, the only significant difference resulted between the samples from zoos without any parasitological management measure vs. all other samples originating from zoos with measures that included anthelmintic treatments and/or faecal examinations before targeted treatments (indifferently from the adopted frequency): the former had a 3.4 higher likelihood (95 % CI: 1.1–10.3) than the latter group to result in positive helminth findings (Table 1).

**Table 1:** Odds ratios (OR) and 95 % confidence intervals (95 % CI) for parasitological management measures in Swiss zoos and wildlife parks associated with a higher probability of detecting helminth stages in carnivore faeces. The management measures were divided in the following categories: 1 = no deworming, no faecal examinations; 2 = deworming frequency <4x/year; 3 = deworming at least 4x/year; 4 = targeted deworming following positive faecal examinations, conducted <4x/year; 5 = targeted deworming following positive faecal examinations carried out 4x/year or more). ∗ indicates a statistically significant p-value.

Comparison of different parasitological management measures in relation to positive helminth findings	OR	95 % CI	p-value
Samples from zoos without (category 1) vs. samples from zoos with any parasitological management measure (categories 2, 3, 4, 5)	3.4	1.1–10.3	0.03∗
Samples from zoos that adopted anthelmintic treatments (categories 2, 3) vs. samples from zoos that adopted faecal examinations followed by targeted deworming (categories 4, 5), independent of the frequency of the adopted measures	1.3	0.8–2.3	0.31
Samples from zoos without deworming management or deworming less than 4x/y or adopting targeted deworming but less than 4x/y (categories 1, 2, 4) vs. samples from zoos implementing either deworming at least 4x/year (category 3) or targeted treatments based on parasitological examinations at the same frequency (category 5)	0.8	0.4–1.4	0.43

Data about animal health status at the point of faecal collection were partially collected in 2018 and from all animals in 2024 (Suppl. files 1, 2). Among these 150 samples, clinical signs were reported from 16 animals: diarrhoea (n = 10), emaciation (n = 4), or nasal discharge and coughing (n = 2). Of these, nine were parasitologically positive, and in 7/16 samples a potential association between clinical finding and positivity was assumed (Suppl. file 2). Overall, 74 samples of animals with no report on clinical signs were coproscopically negative, but 60 samples were parasitologically positive with no report on clinical signs, and 7 samples were parasitologically negative but animals presented with clinical signs. The OR for animals presenting clinical signs vs. animals without notified clinical signs in association with positive parasitological findings were 8.2 (95 % CI: 0.9–76.2, p = 0.07) in 2018 and 0.4 in 2024 (95 % CI: 0.08–2.2, p = 0.30) for positive parasitological findings, indicating a clear investigation bias in 2018.

In 2024, further information about feeding, husbandry, cleaning routine, potential contact with wild animals and past parasitological infections was collected (for details: see Suppl. files 1, 2). Feeding plans varied considerably for the different species. About half of the zoos and wildlife parks mentioned on-site slaughter of zoo and/or farm animals (n = 11). The most frequently administered meat sources were beef, horse, chicken, rabbit, guinea pig, mouse, rat and fish. Water sources varied depending on the enclosures, with tap water being the most common (87/116), followed by spring water (21/116). The setup of the enclosures varied depending on the specific zoo and the animal species. Particularly the natural conditions within the zoos influenced the hygiene measures. For instance, if zoos mentioned the use of soap or disinfectants, this was limited to specific areas within the enclosures (e.g. stables, pools). Zookeepers were asked to estimate the percentage of faeces removed: this varied widely between the different zoos and within the zoos themselves, depending on the enclosure (0–100 %, with an average of 62.0 %; for 15 samples no information was available).

For 41 out of 116 of the sampled animals or animal groups catching birds, rats, mice and or slugs was observed. The analysis of potential risk factors revealed that the likelihood of positive parasitological findings was significantly higher in animals that had the possibility of catching prey animals (i.e., birds, rats, mice, slugs) within their enclosures, with an OR of 2.3 (95 % CI: 1.0–5.0, Table 2). Interestingly, slugs were observed in enclosures of 102 animals and mice in 115. However, there was no significant difference in positive/negative parasitological findings between animals from enclosures where slugs or mice were observed and those where they were not (Table 2).

**Table 2:** Odds ratios (OR) and 95 % confidence intervals (95 % CI) of potential risk factors associated with positive parasitological findings in faecal samples from Swiss zoo carnivores. ∗ indicates a statistically significant p-value.

Potential risk factors	OR	95 % CI	p-value
Observed presence of slugs in enclosures (vs. not observed)	0.4	0.1–1.2	0.10
Observed presence of mice in enclosures (vs. not observed)	1.0	0.2–4.7	0.99
Possibility of catching preys, i.e. birds, rats, mice, slugs (vs. no possibility)	2.3	1.0–5.0	0.04∗
On-site slaughter of zoo and/or farm animals (vs. no on-site slaughter)	0.5	0.2–1.1	0.09
Usage of spring water (vs. tap water)	1.0	0.4–2.6	0.98

### 3.2 Faecal examinations

**Fig. 2:**
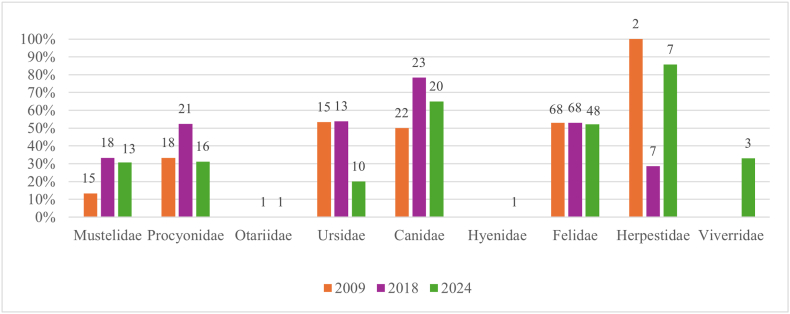
Number (above columns) and percentage of endoparasite positive samples in the nine examined carnivore families per study year.

**Fig. 3:**
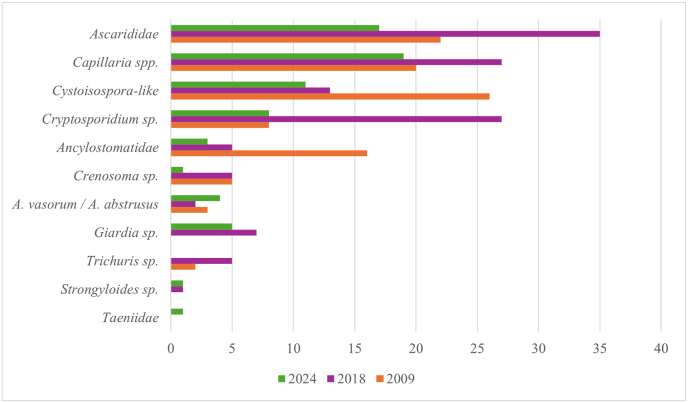
Number of endoparasite positive samples/year in descending frequency of parasite species or family.

The overall percentage of positive samples was 48.9 % (CI: 43.9–53.9 %), decreasing from 52.3 % in 2009 (CI: 41.4–58.6 %) to 43.1 % in 2024 (CI: 33.9–52.6 %). The percentage of positive samples varied between the examined taxonomic families (Fig. 2, Suppl. File 1, 3). The most commonly identified parasites were ascarids (n = 73, 18.0 %), followed by *Capillaria* spp. (n = 66, 16.3 %), *Cystoisospora* spp. (n = 50, 12.3 %) and *Cryptosporidium* (n = 43, 10.6 %) (Fig. 3). Fifteen zoos were sampled three times, but the number of positive samples per year was low, not allowing a comparison between the years.

Adult worms present in faeces were reported to be found in seven cases and identified as *Toxascaris leonina* (lion), *Ascaris* sp. (brown bear), *Capillaria* sp. (brown bear) and *T. cati* (snow leopard) where listed.

#### 3.2.1 Mustelidae

Overall, 46 samples of members of Mustelidae family were analysed: 15 in 2009, 18 in 2018 and 13 in 2024. This included the following animal species: Asian small claw otter (*Aonyx cinereus*, n = 12), European otter (*Lutra lutra*, n = 14), beech marten (*Martes foina*, n = 6), European pine marten (*Martes martes*, n = 10), European badger (*Meles meles*, n = 4), ferret (*Mustela furo*, n = 4), polecat (*Mustela putorius*, n = 2), striped skunk (*Mephitis mephitis*, n = 1), wolverine (*Gulo gulo*, n = 1) and tayra (*Eira barbara*, n = 1).

In 76.1 % (n = 35) of all examined samples, no parasitic stages were identified. The most frequently detected parasites were *Capillaria* spp. (n = 6, 13.0 %) and *Cryptosporidium* sp. (n = 5, 10.9 %). Further samples were positive for Ancylostomatidae (n = 2), and two samples for ascarids and one for *Crenosoma* sp. One of the ascarid samples was identified as *Baylisascaris columnaris* (sequence: 96.6 % identity, 84/87 bp, Table 3).

**Table 3:** Sequencing of four copromicroscopic positive samples further analysed by PCR.

Sample ID	Species	Targeted gene	%	bp	GenBank	Parasite
WK Tae	European wild cat	12S (rnS)	99.5	213/214	EU219554	*Taenia taeniaeformis*
Sk Bay	Stripped skunk	cox2	96.6	84/87	MH469663	*Baylisascaris columnaris*
WK Str	European wild cat	18S RNA	99.4	158/159	JX674037	*Rhabditophanes* sp.
ABB Cre	American black bear	18S RNA	97.6	746/764	AB923889	*Strongyloides ratti*

The percentage of positive samples among all Mustelidae species was compared, identifying a significant difference (Chi-square test p = 0.01), with all beech martens, European badgers, polecats and European pine marten being positive, in opposition to the Asian small claw otters, ferrets, tayra, striped skunk, and wolverine being negative. Two Mustelidae samples originated from animals showing clinical signs: a European badger (*Meles meles*) positive for *Capillaria* spp. and *Cryptosporidium* showed a reduced body condition score, while a European otter (*Lutra lutra*) with diarrhoea was parasitologically negative.

#### 3.2.2 Procyonidae

A total of 49 Procyonidae samples were analysed (16, 20 and 13 in the 3 study periods, respectively). These samples included raccoons (*Procyon* spp., n = 40), coatis (*Nasua sp.*, n = 8) and kinkajous (*Potos flavus*, n = 1).

The most frequently identified parasite genus was *Capillaria* sp. with 14.3 % (n = 7, all from raccoons), followed by *Cryptosporidium* sp. with 12.2 %(n = 6). Ascarids were less common, identified in four samples (n = 3 *Ascaris* sp., n = 1 *Baylisascaris* sp.), *Cystoisospora* sp., identified in three samples, and Ancylostomatidae*,* and *Trichuris* spp. in two samples each. No significant difference in the infection rate between the three different species was observed (Chi-square test p = 0.61). Overall, 29 samples (59.2 %) were negative in all four methods. A single raccoon (*Procyon lotor*) with weight loss was diagnosed positive for *Cryptosporidium* oocyst excretion.

#### 3.2.3 Ursidae

The 38 samples (15, 13 and 10 in the 3 study years, respectively) of Ursidae originated from the following eight animal species: Eurasian brown bear (*Ursus arctos horribilis*, n = 10), Syrian brown bear (*Ursus arctos syriacus*, n = 3), Ussuri brown bear (*Ursus arctos lasiotus*, n = 2), spectacled bear (*Tremarctos ornatus*, n = 5), polar bear (*Ursus maritimus*, n = 1), sun bear (*Helarctos malayanus*, n = 2), American black bear (*Ursus americanus*, n = 4) and Asian black bear (*Ursus thibetanus*, n = 1).

The most common parasites were *Crenosoma* sp., *Cryptosporidium* and ascarids (each n = 5, 13.2 %), closely followed by *Capillaria* sp. (four positive samples, 10.5 %). Among the five ascarid samples, *Baylisascaris* sp. was suspected in one, but not confirmed by genetic methods. Ancylostomatidae (n = 2), *Trichuris* sp., *Strongyloides* sp. and *Giardia* (each n = 1) were less common. No significant difference in infection rate of the different Ursidae species was observed (Chi-square test p = 0.64). Of all examined samples, 21 samples (55.3 %) were negative.

Interestingly, DNA isolated from a larva from an American black bear in 2018 and microscopically suspected to be *Crenosoma* sp. was molecularly identified as *Strongyloides callosciurus* (Table 1).

#### 3.2.4 Canidae

In total, 65 Canidae samples (22, 23 and 20 in 2009, 2018 and 2024, respectively) originating from seven species were analysed: artic wolf (*Canis lupus arctos*, n = 2), European wolf (*Canis lupus lupus*, n = 25), African wild dog (*Lycaon pictus*, n = 3), raccoon dog (*Nyctereutes*, n = 7), arctic fox (*Vulpes lagopus*, n = 5), red fox (*Vulpes vulpes*, n = 21) and fennec fox (*Vulpes zerda*, n = 2).

**Fig. 4:**
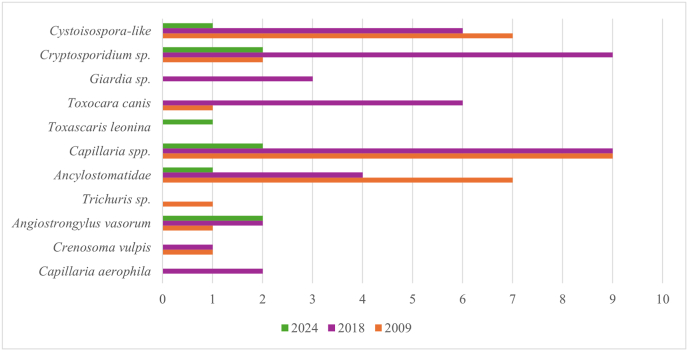
Endoparasitic infections detected in Canidae (n = 65): number of samples positive for intestinal protozoa and helminths and cardiopulmonary parasites determined in the three study periods.

The most frequently identified parasite genus was *Capillaria* sp., with 26 positive samples (40.0 %, 95 % CI: 28.0 %–52.9 %) (Fig. 4). Morphological criteria were used to differentiate between *Capillaria* species: in five samples eggs were identified as *C. aerophila* (syn. *Eucoleus aerophilus*), and in one sample as *C. boehmi* (syn. *Eucoleus boehmi*). The second most common parasites were *Cryptosporidium* and *Cystoisospora* spp., with 13 positive samples each (20.0 %, 95 % CI: 11.1 %–31.8 %), closely followed by Ancylostomatidae with 12 positive samples (18.5 %, 95 % CI: 9.9 %–30.0 %). In addition, *Toxocara canis* (n = 7), *Angiostrongylus vasorum* (n = 5), *Giardia* sp. (n = 3), *Crenosoma vulpis* (n = 2), *Toxascaris leonina* (n = 1) and *Trichuris* spp. (n = 1) were identified.

The percentage of endoparasite positive samples in Canidae species varied over the three study periods, but without a significant difference (see overlapping 95 % CI, Suppl. file 3). *Capillaria* spp. infection varied between 40.9 %, 47.8 % and 30.0 % in the three study years, respectively. In 2009 and 2024, *Cryptosporidium* infection rates were comparable with 4.6 % and 10.0 % respectively, whereas in 2018 the rate was higher (43.5 %). In 2009 and 2018 31.8 % and 26.1 % of samples were positive for *Cystoisospora* spp., respectively, with none of the samples being positive in 2024. Furthermore, 31.8 %, 17.4 % and 5.0 % of the samples were positive for Ancylostomatidae in 2009, 2018 and 2024, respectively.

There was no significant difference in prevalence of endoparasite infection between the examined Canidae species (Fisher exact test p = 0.07). Overall, 23/65 samples (35.4 %) were negative with all four coproscopic methods. Four out of five samples positive for *A. vasorum* collected in 2018 or 2024 were confirmed by a corresponding band in the species-specific PCR.

Overall, clinical signs were reported in association to three Canidae samples (Suppl. files 1, 2). A red fox (*Vulpes vulpes*) positive for six parasite species (i.e., hookworms, *T. canis, Cystoisospora* sp., *Capillaria* sp., *A. vasorum*, and *Cryptosporidium*) showed emaciation, possibly correlated with its cardiopulmonary (*A. vasorum*) and intestinal infections. A European wolf (*Canis lupus lupus*) with diarrhoea was identified positive for hookworms, *Capillaria* sp. and *A. vasorum*. Furthermore, an arctic fox (*Vulpes lagopus*) that was diagnosed positive for *C*. *boehmi*, a parasite of the respiratory airways, showed coughing and choking.

#### 3.2.5 Felidae

Overall 184 Felidae samples (n = 68 in 2009, n = 68 in 2018, n = 48 in 2024) from 14 different animal species were analysed: cheetah (*Acinonyx jubatus*, n = 16), caracal (*Caracal caracal*, n = 2), European wildcat (*Felis silvestris*, n = 18), serval (*Leptailurus serval serval*, n = 8), lynx (*Lynx* spp., n = 41), tiger (*Panthera tigris*) (n = 30), snow leopard (*Panthera uncia*, n = 13), lion (*Panthera leo*, n = 25), leopard (*Panthera pardus*, n = 16), puma (*Puma concolor*, n = 11) and one sample each of pallas's cat (*Otocolobus manual*), Asian leopard cat (*Prionailurus bengalensis*), jungle cat (*Felis chaus*) and ocelot (*Leopardus pardalis*).

**Fig. 5:**
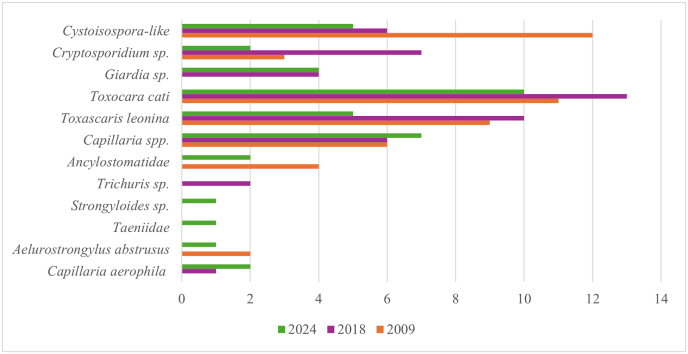
Endoparasitic infections detected in Felidae (n = 184): number of samples positive for intestinal protozoa and helminths and lungworms determined in the three study periods.

The most frequently identified parasite was *Toxocara cati,* with eggs detected in 34 samples (18.5 %, s95 % CI: 13.2 %–24.9 %) (Fig. 5). This was followed by *Toxascaris leonina* with 24 positive samples (13.0 %, 95 % CI: 8.5 %–18.8 %). Accordingly, ascarids were identified in 31.5 % of the felid samples. *Cystoisospora* spp. oocysts were found in 23 samples (12.5 %, 95 % CI: 8.1 %–18.2 %) and *Capillaria* eggs in 22 cases (12.0 %, 95 % CI: 7.7 %–17.5 %). Of these, three were morphologically identified as *C. aerophila*. Furthermore, *Cryptosporidium* cysts were found in 12 samples. The following parasites were additionally identified: *Giardia* sp. (8 antigen positive samples), hookworm eggs (n=6), *Aelurostronglyus abstrusus* first stage larvae (n = 3), *Trichuris* spp. (2), *Strongyloides* spp. eggs and first and third stage larvae (n = 1), and taeniid eggs (n = 1).

Comparing the study periods, no significant changes in parasite prevalence were identified (Fig. 5). As an example, *T. cati* was found in 16.2 %, 19.1 % and 20.8 %, and *T. leonina* in 13.2 %, 14.7 % and 10.5 % in the respective three study years. In contrast, infection rates by Felidae varied significantly between the different examined species (Chi-square test p < 0.001). Of all samples, 86/164 samples (46.7 %) were negative in all four coproscopic methods.

The positive taeniid sample of a European wildcat collected in 2024 was positive in the multiplex PCR. The sequence showed a 99.5 % identity (213/214 bp) to *Taenia taeniaeformis* (Table 3). The presence of *A*. *abstrusus* L1 in a faecal sample collected in 2014 from a cheetah was further confirmed by the respective specific band present in the duplex PCR. The single sample of a European wildcat morphologically positive in 2024 for *Strongyloides* sp. could not be molecularly confirmed (blast sequencing showed a 99.4 % identity with a genus belonging to the free-living nematodes (*Rabditophane*s sp., Table 3).

Overall, 8 Felidae samples originated from animals showing clinical signs. A European wild cat (*Felis silvestris*) was positive for intestinal protozoan *Cryptosporidium* oocysts but showed nasal discharge. A snow leopard (*Panthera uncia*) was positive for *Toxascaris leonina* and observed to have mild diarrhoea. Similarly, a tiger (*Panthera tigris*) with mild diarrhoea was positive for *Toxocara cati.* In contrast, a snow leopard showing emaciation, an Asian leopard (*Prionailurus bengalensis*) with mild diarrhoea and two serval (*Leptailurus serval serval*) and a chetaah (*Acinonyx jubatus*) also with diarrhoea were all parasitologically negative (the latter animal was diagnosed positive for *Campylobacter*, oral communication).

#### 3.2.6 Herpestidae

A total of 16 samples of animals of the Herpestidae family were analysed: 2, 7 and 7 in the 3 study years respectively, originating from the three species, i.e., dwarf mongoose (*Helogale parvula*, n = 2), yellow mongoose (*Cynictis penicillate*, n = 2) and meerkat (*Suricata suricatta*, n = 12). Only meerkats harboured parasitic stages, and overall, six out of 16 samples (37.5 %) were coproscopically negative. The most frequently identified parasitic stages were *Cystoisospora*-like oocysts, being present in 7 meerkats (43.8 %). The occurrence of positive *Cystoisospora*-like samples varied over the years between one and four. In addition, the following parasites were identified in one sample each: *A. vasorum*, *Capillaria* sp., *Crenosoma* sp., *Trichuris* sp. and *Cryptosporidium*.

#### 3.2.7 Ailuridae, Otariidae, Hyaenidae, Viverridae

In the three study years a total of six Ailuridae samples were analysed. In the first two years, two samples were positive for *Crenosoma* spp. Furthermore, one sample was found to be positive for *Metastrongylus* sp. In 2024 all three samples were negative. Two samples of sea lions (*Zalophus californianus*, Otariidae), one collected in 2018 and one in 2024, were coproscopically negative. A single sample originating from Hyaenidae was analysed in 2024: a spotted hyena (*Crocuta*) was negative in all four coproscopic methods. Three samples originated from Viverridae species: two from fossae (*Cryptoprocta ferox*) and one from a genet (*Viverra genetta*), all collected in 2024. The two fossae samples were negative (but showing intermittent diarrhoea), whereas the genet was positive for *Cystoisospora* sp.

## 4 Discussion

In the three years of investigations, 405 faecal samples originating from 50 carnivore species housed in 30 institutions were examined for endoparasites, providing a comprehensive overview of the endoparasitic load in zoo-housed carnivores in Switzerland. The large number of animal species examined entails a wide range of behaviours and husbandry requirements, addressed by highly variable enclosure designs and management strategies, likely mirroring the broad spectrum of zoo-housed carnivore practices worldwide. Although the study period covered 15 years, the differences between the three study years in terms of percentages of positive animals and the nature of parasitological findings were minor. A major limitation is the lack of systematically recorded clinical signs (particularly in 2009 and 2018), so that clinical findings were only recorded for a total of 16 animals. Among these, a potential correlation with parasitological findings was hypothesised in seven cases. Most samples were obtained from group housings, and, therefore, attribution to individual animals was only possible in few cases. Also, alternative aetiologies contributing to the clinical manifestations cannot be excluded. On a positive note, approximately half of the examined animals/animal groups were parasitologically negative and, where positive, the parasitic infections were mostly not associated with clinical signs. However, the performed examinations only reflect a specific moment in time, and infections were not quantitatively assessed in our study. Hence, the parasitic burdens could have been minor, and their effects subclinical.

Information on the adopted parasite management as well as on potential risk factors was investigated in 2024 and partially also in 2018 (deworming strategy). Although an increasing number of zoos (21.6 % in 2024 vs. 13.1 % in 2018) performed preventive measures (i.e., at least four faecal examinations per year followed by targeted deworming according to findings), no significant decrease of overall parasite infections was observed. This may partly reflect the persistent reinfection risk from environmental parasite stages or intermediate hosts. The evaluation of potential enclosure-related risk factors revealed that the possibility for animals to prey on birds, rodents, or slugs was significantly associated with a higher likelihood of positive parasitological findings, whereas on-site slaughter appeared to have a protective effect by trend.

In carnivore companion animals, namely dogs and cats, parasitological control strategies are designed to safeguard animal health and reduce the risk of zoonotic parasite transmission. Guidelines established for domestic companion animal carnivores (i.e. from ESCCAP - European Scientific Counsel Companion Animals, or CAPC – Companion Animal Parasite Council) recommend regular deworming or faecal examinations followed by targeted treatments in frequencies on individual risk assessments that consider the possibility of the animal to have regular outdoor access without supervision and therefore to ingest infected prey animals or gastropods (www.esccap.org, Guideline no. 1 – Worm control in dogs and cats). In opposition to livestock, anthelmintic resistance remains rare but is primarily anticipated in settings such as kennels and catteries, where limited space, high animal density, and frequent deworming may promote selection pressure (von Samson-Himmelstjerna et al., 2021). These conditions could be comparable to those present in carnivore housing within zoological institutions. Therefore, we aimed to investigate whether blind deworming would result in more positive parasitological findings compared to targeted deworming following parasitological examination: this was not confirmed. No information was available on the presence of potentially resistant isolates that might persist despite deworming: to assess this, follow-up examinations after anthelmintic treatments would be recommended. Interestingly, there was also no significant difference between animals that were dewormed or that were examined and subjected to targeted deworming fewer than four times a year vs. animals that were either dewormed or examined and dewormed in a targeted manner at least four times a year. The highest and significant likelihood for positive parasitological findings was obtained for samples originating from zoos that did not take any measures compared to zoos that had some kind of deworming strategy.

The specific parasitological findings are discussed in the following, as they may become significant in case of chronicity (Collett et al., 2000; Polotzek et al., 2025), of the presence of young animals (Roller et al., 2021) or in animals with immunosuppression and/or stress (Andre et al., 2010; Colly and Nesbit, 1992) or with concomitant diseases (Munson et al., 2008).

### 4.1 Ascarids

The most common parasites detected in all families were ascarids, detected in 19.0 % (77/405) of the samples. This aligns with a recent overview from German zoos (16.6 %) (Murnik et al., 2024), although ascarid prevalence may be up to 40.0 %, 66.7 %, or 57.1 % in Europe (Fagiolini et al., 2010; Maesano et al., 2014; Okulewicz et al., 2002) and worldwide (64.3 %) (Lim et al., 2008).

*Toxocara canis* is also the most frequent nematode in domestic dogs because it is very efficiently transmitted via the placenta or via milk, or directly through oral egg ingestion from the environment or through a paratenic host, whereas *T. cati* is not transmitted prenatally (Deplazes et al., 2016; Parsons, 1987). In domestic cats, especially in stray animals, *Toxocara* prevalence is high, with 55 % of stray cats excreting *T. cati* eggs in Switzerland (Zottler et al., 2019). The percentage of positive ascarid samples was highest in Felidae, with 31.5 %: of these, 24 (41.4 %) were positive for *T. leonina* and 34 (58.6 %) for *T. cati.* In Canidae, 10.8 % of the samples were *T. canis* positive (and only 1.5 % were *T. leonina* positive).

*Toxocara* spp. infections can impact animal health and increase their susceptibility to other infectious diseases (Deplazes et al., 2016). Newborn animals are at risk for heavy and potentially clinically relevant infections. Post-mortem examinations of captive red wolves (*Canis rufus*) revealed internal parasitism in 86 % of neonates, with eosinophilic pneumonia and enteritis attributed to migrating stages of *Toxocara* larvae, and pneumonia was identified as the predominant cause of death (Acton et al., 2000). In our study, most of the affected canids and felids were asymptomatic, which aligns with other studies on zoo carnivores (Fagiolini et al., 2010; Maesano et al., 2014). Possibly the positive but asymptomatic animals may have had a low parasitic load. However, among the few animals reported with clinical signs and being positive for ascarids, there was one red fox and two felids showing signs compatible with an intestinal infection. In zoo carnivores, the prevention of neonatal transmission is particularly challenging. A treatment protocol with fenbendazole administered daily to pregnant females from day 40 of gestation until 2 weeks post-partum, comparable with female dogs, was recommended (Acton et al., 2000). Although *Toxocara* eggs are not directly infectious after excretion, their thick and chemically resistant wall allows long-lasting persistence (up to four years) in the environment, and infections may be seen even in adult animals. In contrast, *T. leonina* infections in felids and canids are mostly subclinical, and eggs do not have zoonotic potential.

Ascarids were also detected in samples of Mustelidae, Procyonidae and Ursidae. Even though prevalence was low in these animal species and there were no ascarid positive animals reported with clinical signs, the zoonotic potential of ascarids needs to be considered when processing positive faecal samples. In particular, *Baylisascaris procyonis* is known for causing severe disease that can induce meningoencephalitis or neuroretinitis in zoo-housed birds (Thompson et al., 2008), porcupines (Fitzgerald et al., 1991), in a silver fox (Larson and Greve, 1983) and in humans due to neural and ocular larva migrans (Goldberg et al., 1993; Kazacos et al., 2013). Here, *B. columnaris* was diagnosed in a striped skunk, and although the most pathogenic species is *B. procyonis*, this parasite is also a potential cause of visceral, ocular or neural larva migrans in humans (D'Ovidio et al., 2017).

Appropriate hygiene measures (i.e., wearing gloves, safe disposal of faeces) when handling faecal samples of carnivores are therefore relevant in the context of human toxocariasis. The diagnosis of toxocariasis in humans is challenging because antibodies directed against *Toxocara* sp. may persist for years, and a correlation with clinically relevant signs needs to be supported with further diagnostic procedures. When diagnosing ascarids, anthelmintic treatments should be combined with further measures such as environmental hygiene, with elimination of potentially infectious eggs, and health checks of involved animal keeper personnel.

### 4.2 Cardiopulmonary nematodes

Metastronyloid cardiopulmonary nematodes were identified in several carnivore samples in this study. *Angiostrongylus vasorum* was confirmed in one sample of meerkats, in two of European wolves and in three red fox samples. Furthermore, one Cheetah was infected with *A. abstrusus*, and in six samples of Ursidae, one of polecats and one of red pandas, *Crenosoma* sp. was identified.

Infections with metastrongyloids were repeatedly observed in red pandas (*Ailurus fulgens*). In a large study conducted with 115 red pandas from 54 European zoos, 35 % of the animals were shedding L1, with overall 37 % of the animals excreting non-identified L1, 4.4 % shedding *Crenosoma* and 2.4 % *A. vasorum* L1 (Bertelsen et al., 2010). Infections with *Crenosoma* sp. are also frequently identified in Ursidae, and in single cases the clinical relevance was ascertained in animals in the wild (Mahjoub et al., 2020).

The occurrence of both *A. vasorum* in canid and *A. abstrusus* in felid samples in zoo animals of Switzerland is not surprising: these parasites regularly infect domestic dogs and cats, respectively. Wild foxes represent the main reservoir hosts for *A. vasorum* and stray cats for *A. abstrusus*, with regional prevalences above 80 resp. 20 %, contributing to the contamination of surroundings (Gillis-Germitsch et al., 2020b; Gueldner et al., 2019). The life cycle of these parasites includes an obligate intermediate gastropod host. Accordingly, with territories surrounding zoo and animal parks allowing the presence of foxes and cats, and because it is virtually impossible to prevent snails from accessing the enclosures, infectious stages may be ingested by zoo carnivores through the consumption of gastropods. This is exemplified in *A. vasorum* infections of 7 out of 17 meerkats kept in a research enclosure at the University of Zurich, Switzerland: in their immediate surrounding 2.1 % of gastropods were also positive (Gillis-Germitsch et al., 2017). Dogs are the main domestic host and fatal cases are regularly described (Chapman et al., 2004; Glaus et al., 2016; Sigrist et al., 2017). Due to low host specificity of *A. vasorum* affecting also Herpestidae, Ailuridae (i.e., red pandas) (Bertelsen et al., 2010; Patterson-Kane et al., 2009) and Mustelidae (i.e., stoats, weasels, badgers and otters) (Madsen et al., 1999; Simpson, 2010; Torres et al., 2001), clinically relevant infections need to be considered in zoo animals (Bagardi et al., 2021; Patterson-Kane et al., 2009), because they may lead to potential fatal outcomes (M. Kummrow, personal communication). In the present study, a single red fox harbouring *A. vasorum* (together with five other parasite species) showed evident clinical signs, i.e. emaciation, which may be compatible with the impressive compilation of parasitological findings.

In domestic cats, anaesthesia combined with verminous pneumonia was associated with death in most of the cases where the cause could be identified (Gerdin et al., 2011). Accordingly, lungworm infections should not be underestimated in zoo carnivores, particularly if they undergo anaesthesia.

Virtually all enclosures of the sampled animals were at least partially outdoors, replicating natural ecosystems with trees and natural soil; therefore, once present, the eradication of infected intermediate hosts will most likely not be possible. Despite the overall low number of lungworm-positive samples, the potential major impact of these parasites on animal health highlights the need for preventive measures, such as regular screening of susceptible animal species and anthelmintic treatment in case of positive findings.

### 4.3 Trichuridae

The transmission of *Capillaria* sp. depends on the *Capillaria* species itself, with direct as well as, in some cases, indirect transmission (intermediate host: earthworms) (Deplazes et al., 2016). *Capillaria putorii* infects Mustelidae, Felidae, and Procyonidae, whereas *C*. *aerophila* was detected in Mustelidae, Felidae as well as in Canidae (Deplazes et al., 2016), but their clinical relevance in zoo animals is unclear. We identified *Capillaria* spp. in 61/405 of the examined samples and in 19 different carnivore species. However, many of the zoos mentioned feeding fresh poultry products and other birds and wild animals (mice and rats) present in many enclosures, all representing sources of other morphologically indistinguishable *Capillaria* sp. eggs. In these cases, intestinal passages cannot be ruled out.

Most animals were asymptomatic. A single arctic fox infected with *Capillaria boehmi* was coughing and choking, similar to clinical signs described in infected dogs (Gillis-Germitsch et al., 2020a; Traversa et al., 2010). Recently, four out of five zoo-housed arctic foxes presenting with coughing and further respiratory signs died or were euthanised due to fatal pneumonia associated with *C. aerophila* infections (Polotzek et al., 2025). The anthelmintic elimination for both, *C. aerophila* and *C. boehmi*, represents a major challenge, as classical protocols for intestinal helminths are insufficient (Gillis-Germitsch et al., 2020a; Polotzek et al., 2025). Moreover, the long-term persistence of infective *Capillaria* eggs in the environment, together with transmission through intermediate hosts in natural enclosures, significantly complicates eradication efforts.

*Trichuris* spp. eggs were identified in 7 samples from 6 different animal species belonging to 5 families. No impact on animals was observed, confirming their limited relevance for zoo carnivora. A minimal risk of zoonotic transmission to people handling faecal samples has to be considered (Deplazes et al., 2016).

### 4.4 Protozoa

One quarter of the analysed samples (101/405) were positive for intestinal protozoan stages. The most frequently detected protozoans were *Cystoisospora* sp. or *Cystoisospora*-like shaped oocysts, present in 12.1 % of the animals, followed by *Cryptosporidium* oocysts (9.9 %) and *Giardia* antigen detection (3.0 %). Despite their wide occurrence, their clinical relevance is generally limited to juvenile animals (Deplazes et al., 2016) that may present with diarrhoea, anorexia and dehydration (Meny et al., 2012).

Particularly family groups with juveniles may present higher rates of infection. Accordingly, the percentage of meerkats positive for *Cystoisospora* was particularly high (7/12), however, it had no clinical impact. Only a single red fox that was harbouring also *T. canis, A. vasorum* and 4 other parasite species, including *Cystoisospora,* showed a reduced body condition score.

*Cryptosporidium* sp. has been regularly detected in zoo carnivores, between 0.1 % and 7.2 % (da Silva Barbosa et al., 2020; Karim et al., 2021; Matsubayashi et al., 2005; Murnik et al., 2024). Limitations in the interpretation of such data are represented by the challenging detection methods and especially by the lack of differentiation from innocuous intestinal passages caused by feeding fresh meat, for instance veal and chicken, which may contain *Cryptosporidium* stages that cannot be morphologically differentiated. In our study, *Cryptosporidium* sp. was detected in three animals having clinical signs like emaciation, but no evident signs of diarrhoea, as one would expect. No further molecular analyses were performed; however, zoonotic *C. parvum* isolates were previously identified in several wild carnivore species and in zoo carnivores (Matas-Mendez et al., 2024).

The detected percentage of *Giardia* positive samples is comparable to other studies, and very few or no clinical signs in affected animals were reported previously (da Silva Barbosa et al., 2020; Zou et al., 2022). As for domestic dogs and cats, *Giardia* infections may be mostly inapparent, and the clinical relevance of *Giardia* in zoo animals can be questioned. However, although not examined in this study, zoonotic *Giardia duodenalis* assemblages (A and B) have been repeatedly identified in zoo carnivores (Beck et al., 2011; Karim et al., 2021; Matas-Mendez et al., 2024; Zou et al., 2022).

Thus, for both *Cryptosporidium* and *Giardia* sp., close contact with infected animals or their faeces may entail a risk of zoonotic transmission and should be approached with caution (Matas-Mendez et al., 2024). Moreover, in line with observations in domestic animals, the limited availability of effective therapeutic agents poses a major obstacle to successful treatment and control.

Modern zoos prioritise animal welfare management and aim to reduce stress by improving the quality of life of hosted animals. Therefore, environments are enriched with stimuli intended to support naturalistic behaviours (Roe et al., 2014). For carnivores, this includes foraging and hunting on natural soil and hence with access to mice, birds or slugs, implying that parasitic infections with them as intermediate and/or paratenic hosts cannot be excluded. Importantly, in opposition to most domestic dogs and cats with outdoor access, the enclosures of zoo carnivores are limited to the same areas for years or decades, and depending on the hygiene and management measures, infectious parasitic stages may accumulate in the environment or in paratenic or intermediate hosts and therefore represent a continuous infective source. Risk-assessed targeted deworming includes evaluating transmission pathways, and therefore the biological life cycle of every single parasite, and must be linked to information on the parasite's impact on animal health, its zoonotic potential and the practical means of control through the implementation of preventive measures. Accordingly, sustainable parasite control aims for i) limiting infections with clinically relevant parasites, and ii) preventing zoonotic transmission. In relation to i), our results indicate that the implementation of appropriate parasite control and/or monitoring measures is relevant for animals that prey on birds, rodents, or slugs. With regard to ii), transmission to visitors from carnivores in captivity can be excluded, but zoo staff may be exposed during cleaning and handling procedures: therefore, appropriate management of fecal matter and personal hygiene are fundamental, supported by regular animal health assessments and a well-structured staff health program - including training and periodic monitoring - can effectively reduce transmission risks between animals and staff (Esteban-Sánchez et al., 2024).

Ascarids are the most frequently detected helminths and are relevant in terms of both aspects. The prepatency of *T. canis* and *T. cati* being approximately 1–2 months, faecal examinations or deworming should be performed at least four times a year by accepting the risk of egg contamination of the environment. With this procedure, also hookworm or *Trichuris* infections are covered if detection is followed by appropriate anthelmintic treatment. The same applies for the prevention against cardiorespiratory parasites. However, due to the high clinical relevance of *A. vasorum*, even monthly controls may be considered, especially in enclosures with known occurrences of the parasite and where animals are known to consume gastropods. In the specific case of *C. boehmi* and *C. aerophila* detection, the accumulation of infective stages in the environment and difficulties in identifying efficacious anthelmintics can lead to animals presenting with nasal discharge and respiratory issues. For Felidae, verminous pneumonia in combination with anaesthesia was shown to represent a deadly risk factor (Gerdin et al., 2011), and this may be extended to all carnivores that undergo anaesthesia.

In our study, a single sample (European wild cat) was positive for taeniids. Importantly, infections with the highly zoonotic fox tapeworm *Echinococcus multilocularis* should be prevented in Canidae (Deplazes et al., 2011). In Switzerland, this parasite is widely spread in the (sub-)urban fox population (Deplazes et al., 2004), which is the origin of an increased number of human cases of alveolar echinococcosis, but also of deadly cases in zoo-housed primates (Schweiger et al., 2007; Wenker et al., 2019). In domestic dogs, prevention of egg excretion is accomplished by administration of praziquantel, which would represent the treatment of choice also for wild zoo carnivores. Among further parasites with zoonotic potential, *Strongyloides* sp. and *Baylisascaris* sp. should also be considered: although infections are rare, both can lead to severe disease in humans (Bauer, 2011; Jaleta et al., 2017). Regarding protozoa, *Toxoplasma gondii* is the most widely distributed pathogen worldwide, prevalent in virtually all warm-blooded animals, but domestic and wild felids are the only definitive hosts that can excrete oocysts with their faeces (Dubey, 2022). Although *Toxoplasma* oocyst excretion is rare, the contamination of the environment with *Toxoplasma* cysts was at the origin of several deadly cases of toxoplasmosis, i.e. in Australasian marsupials, squirrel monkeys, ring-tailed lemurs, meerkats, Pallas’ cats, certain avian species and many other (Denk et al., 2022; Dubey, 2022). Accordingly, toxoplasmosis prevention in zoo carnivores must focus on preventing the ingestion of small infected intermediate hosts that may occur in the enclosures, together with meat of safe origin that has been frozen at −20 °C for at least 2 days. In our study, the protozoal stages excreted by zoo carnivores were limited to the intestinal protozoa *Cystoisospora, Cryptosporidium* and *Giardia*. Their clinical relevance is minor, and so is the role of zoo carnivores transmitting zoonotic isolates.

## 5 Conclusion

In conclusion, as determining appropriate dosages and routes of administration of antiparasitic treatments can be particularly challenging in zoo animals, emphasis should be placed on preventive approaches. These include rigorous hygiene management, early detection of parasitic infections, and measures to prevent the ingestion of infective stages. Rather than aiming for complete parasite elimination, management efforts may focus on preventing clinical disease, acknowledging that stress or anaesthetic procedures may precipitate severe conditions in valuable individuals. Targeted deworming based on prior parasitological examination is therefore recommended, as it reduces the use of anthelmintics, and consequently limits the development of anthelmintic resistances, the likelihood of adverse effects and decreases stress associated with animal handling.

## CRediT authorship contribution statement

**Jasmin Tan:** Writing – original draft, Investigation, Formal analysis, Data curation, Conceptualization. **Sarah Weber:** Investigation, Data curation. **Ronja Zuber:** Investigation, Data curation. **Maya Kummrow:** Writing – review & editing, Validation, Conceptualization. **Manuela Schnyder:** Writing – review & editing, Writing – original draft, Validation, Supervision, Resources, Project administration, Methodology, Funding acquisition, Data curation, Conceptualization.

## Ethics approval

All applicable international and institutional guidelines for the care and use of animals were followed.

## Financial support

This study was financed by institutional funds.

## Conflict of interest

The authors declare that they do not have conflict of interest to declare.

## Data Availability

The anonymised detailed data are presented in Supplementary Materials:- Supplementary file 1: Master sheet, data from years 2009, 2018, 2024 - Supplementary file 2: Presentation of clinical signs, risk factors, deworming management data and calculations of Odds Ratios. - Supplementary file 3: Results of copromicroscopic examinations per animal species and year.
